# Decreased adhesion to endothelium leads to elevated neutrophil granulocyte count in hereditary angioedema patients

**DOI:** 10.1038/s41598-023-40442-9

**Published:** 2023-08-17

**Authors:** Erika Kajdácsi, Zsuzsanna Balla, Zsófia Pólai, László Cervenak, Henriette Farkas

**Affiliations:** 1 Research Laboratory, Department of Internal Medicine and Haematology, Semmelweis University, Szentkirályi u. 46., Budapest, 1088 Hungary; 2 Hungarian Angioedema Center of Reference and Excellence, Department of Internal Medicine and Haematology, Semmelweis University, Budapest, Hungary

**Keywords:** Cell adhesion, Complement cascade, Cell biology, Immunology, Pathogenesis

## Abstract

As many aspects of hereditary angioedema (HAE) due to C1-inhibitor (C1-INH) deficiency (C1-INH-HAE) cannot be explained with elevated bradykinin level alone, it has recently become clear that other factors also play an important role in the pathogenesis. One of these factors could be elevated neutrophil granulocyte (NG) counts, which are associated with increased NG activation in C1-INH-HAE patients; however, their origin has not been elucidated so far. Here, we aimed to investigate whether the excess of NGs is due to disturbed maturation, biased circulating/marginated pool equilibrium or decreased elimination. We enrolled 20 attack-free C1-INH-HAE patients together with 21 healthy controls and collected blood samples. We compared cell surface maturation markers, adhesion molecules, cytokine receptors, and Ca^2+^-mobilization of NG by flow cytometry, activation markers by ELISA, and NG/endothelial cell adhesion by automated pipetting system. Cell-surface markers showed normal maturation of NGs in C1-INH-HAE patients. Adhesion of NGs to endothelial cells pretreated with lipopolysaccharide or phorbol 12-myristate 13-acetate was significantly weaker in samples from C1-INH-HAE patients and bradykinin had no effect on the adhesion. NGs from C1-INH-HAE patients were in an activated state when assessed by soluble activation markers without any stimulation. Our data support that the maturation of NGs in C1-INH-HAE patients is normal, whereas adhesion properties of patient-derived NGs to the endothelium are reduced compared to those from healthy controls, indicating a bias between the circulating and marginated pools of NGs in patients. Bradykinin may not be responsible for reduced adhesion properties of NGs.

## Introduction

Hereditary angioedem (HAE) a due to C1 inhibitor (C1-INH) deficiency (C1-INH-HAE), a type of bradykinin (BK)-mediated angioedema, is a rare autosomal dominant inherited disorder characterized by intermittent, unpredictable swelling of subcutaneous and/or submucosal tissues. Different mutations in the *SERPING1* gene result in reduced C1-INH production or dysfunctional protein^1^.

C1-INH plays an important role in the regulation of vascular permeability and inflammation. The vascular endothelium has an important role in the pathogenesis of C1-INH-HAE. C1-INH inhibits several plasma serine proteases in classical and lectin complement pathways, intrinsic coagulation (contact system), fibrinolytic and kinin-kallikrein pathways^2^. These plasma enzyme systems are activated in C1-INH deficiency and a number of substances are released that together increases vascular permeability by extravasation of plasma into tissues. BK, generated in the kinin–kallikrein system (KKS), is the main vasoactive mediator^3^. Biochemical processes that take place in plasma are well-studied. Limited data are available on the role of endothelial cells (ECs) and cellular elements in blood during the pathogenesis of C1-INH-HAE, partially because specific in vitro experimental systems would be required to study these cell types.

Previously, our research group found that white blood cell (WBC) count (1.58-fold), and especially neutrophil granulocyte (NG) count (1.95-fold), was significantly higher than expected from hemoconcentration alone calculated from red blood cell count difference^4^. We investigated this phenomenon in a more detailed way as well and found that C1-INH-HAE patients had higher NG levels even in symptom-free state than healthy controls. Furthermore, myeloperoxidase (MPO) and neutrophil elastase (ELA2) measurements showed that NGs in C1-INH-HAE patients in symptom-free state were more activated than those in healthy controls. The activation of NGs was even more significant in prodromal and attack states^5,6^.

NGs are the major leukocyte population in the blood. Their primary function is the rapid and effective phagocytosis of extracellular pathogens, mostly bacteria and fungi, and the subsequent destruction of microbes. Elevated NG counts can be observed in several diseases; however, few pathophysiological processes are directly responsible for neutrophilia. Firstly, more NGs exit the bone marrow in excessive granulopoiesis; however, in this case, neutrophil progenitors can also be found in the circulation, characterized by nuclear morphology (i.e. promyelocyte, myelocyte, metamyelocyte and band forms) and cell surface maturation markers (CD11b, CD13, CD16, and CD66b). The NG count may also be elevated if the rather short normal half-life of neutrophils is somehow prolonged (defect in the programmed cell death machinery, or a defect in the cell killing mechanism, which is frequently associated with neutrophil death). Finally, neutrophilia can be caused by reduced adhesion to ECs, resulting in fewer NGs in the tissues and adherence to vessel walls.

**Figure 1:**
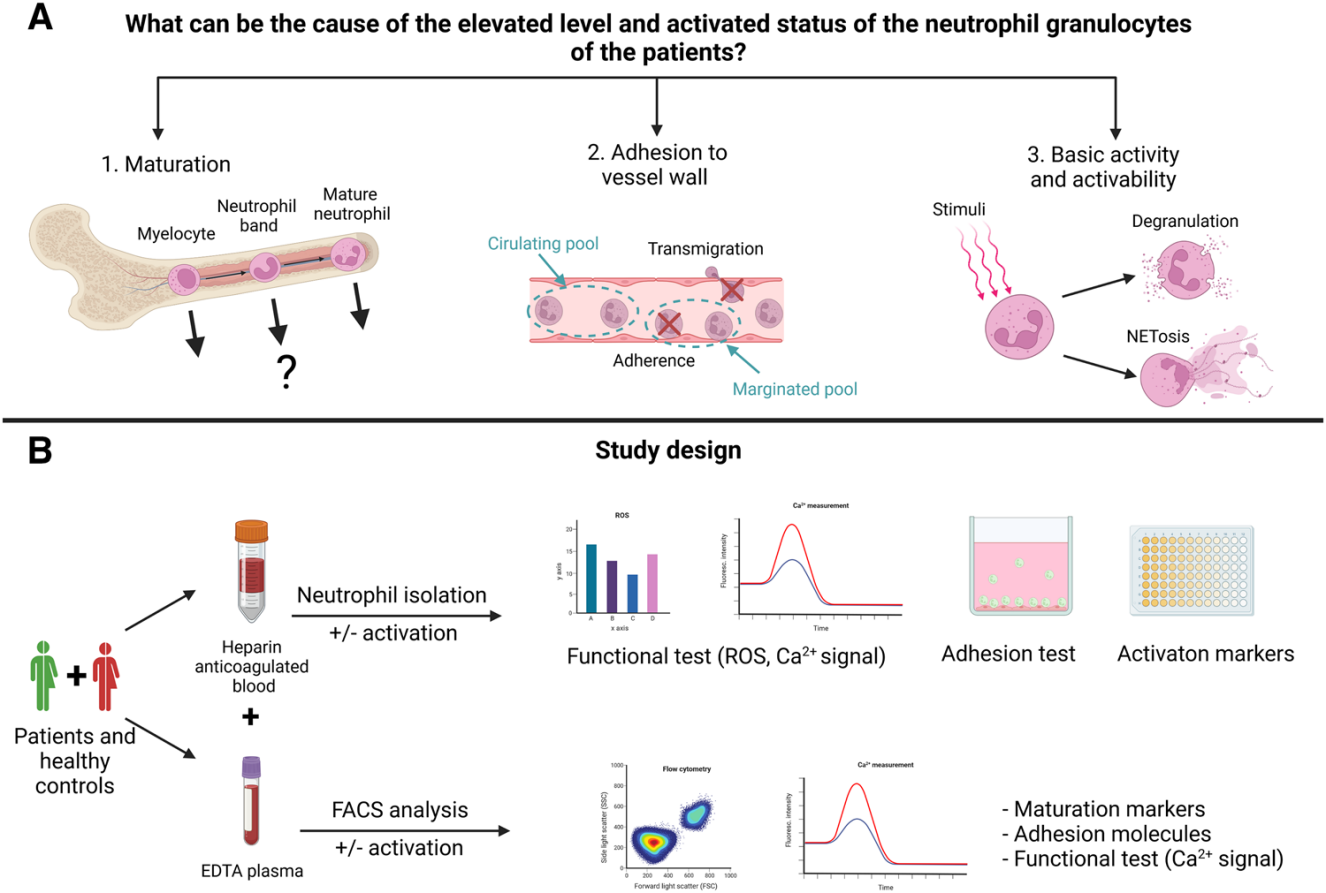
Summary of the study. (**A**) Hypothesis of the study. (**B**) Study design with methods. (created with BioRender.com).

As our starting hypothesis of the current study (visualized in Fig. 1), we tested all possibilities mentioned above. We aimed to investigate the presence of NG progenitors, the expression of adhesion molecules and cytokine receptors, the production of NG effector molecules/systems, such as MPO, neutrophil extracellular trap (NET), ELA2, proteinase 3 (PRTN3) and reactive oxidative species (ROS), intracellular Ca^2+^-mobilization in response to N-formylmethionyl-leucyl-phenylalanine (fMLP) and BK, and the comparison of adhesive characteristics to ECs in C1-INH-HAE patients and healthy controls.

## Results

### Phenotyping of NGs by flow-cytometry

We observed a trend for a higher median of the NG count in C1-INH-HAE samples than in control samples (median and interquartile ranges were 2431 NGs/μl (1764.47; 2909.18) versus 2002 NGs/μl (1756.12; 2522.92), respectively using flow cytometry; however, the difference was not statistically significant (Mann Whitney test p = 0.1035).

To test whether neutrophilia is caused by elevated efflux rate from the bone marrow, we measured NG maturation markers CD11b, CD13, CD16, CD33, CD66b, CD182 and CD184 using flow cytometry. We found no significant differences in the expression of these markers between healthy controls and C1-INH-HAE patients (Supplementary Fig. 1 and Supplementary Table 1).

NGs (after preparation from blood) bind Annexin V, indicating their susceptibility to endocytosis by macrophages. Although NGs from C1-INH-HAE patients bound slightly less Annexin V than NGs from controls (medians of fluorescence intensity (MFI) and interquartile ranges: 18.5 [6.2–39.5] vs. 38.8 [9.3–60.7]), the difference was not statistically significant (*p* = 0.1826, Mann–Whitney test) (Supplementary Fig. 1).

We also assessed the pattern of the most important adhesion molecules and cytokine receptors that regulate neutrophil/endothelial cell or neutrophil/stromal cell adherence. We found a statistically significant but minor difference in the expression of CD61 (Supplementary Fig. 1 and Supplementary Table 1, *p* = 0.0071).

### Functional integrity of the isolated NGs

**Figure 2:**
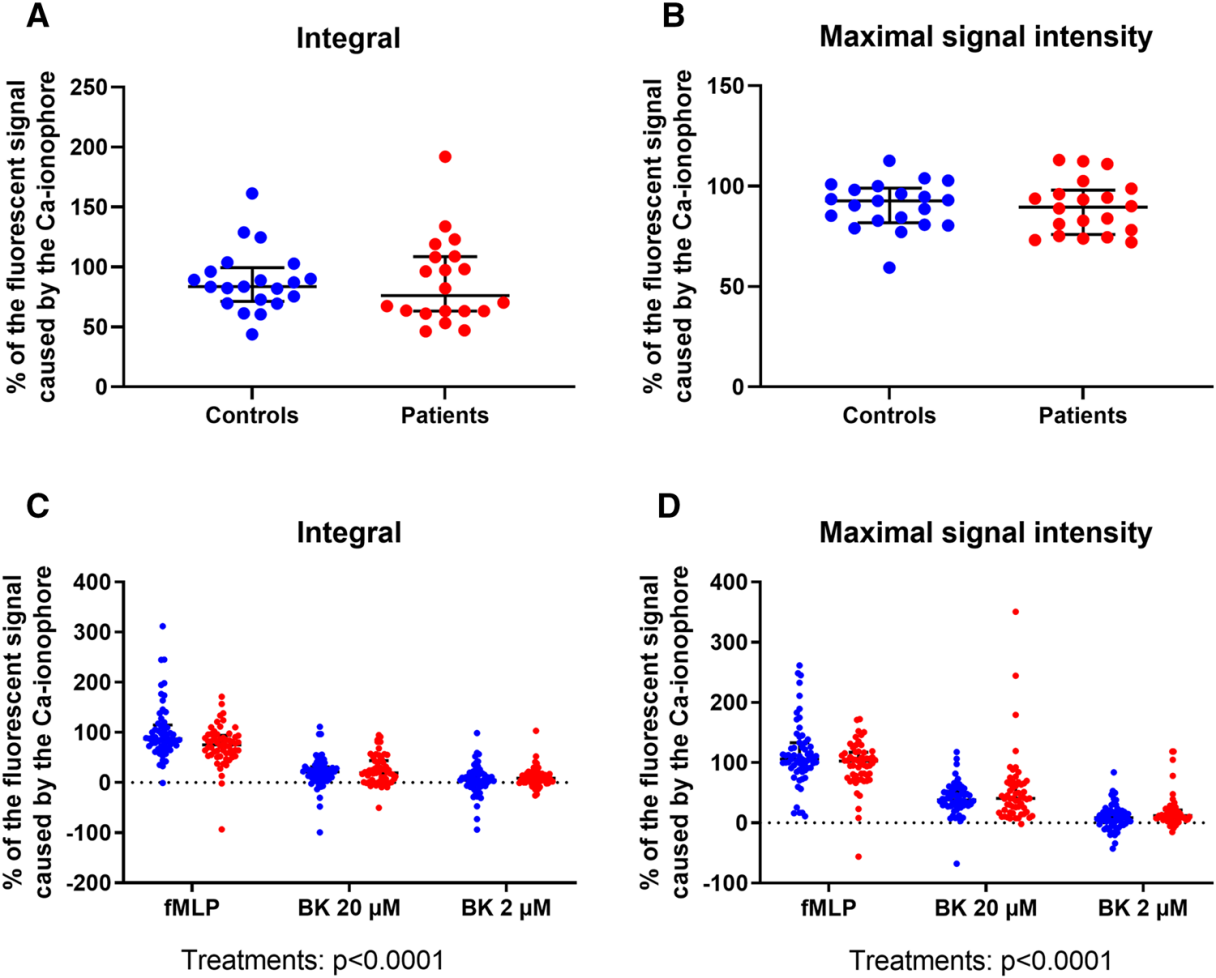
Measurement of intracellular Ca^2+^-mobilization from RBC depleted blood and from isolated NGs. Ca-ionophore was used as a positive control, and the fluorescence signal induced by other agents was expressed as the percentage of the fluorescence signal induced by Ca-ionophore. An aliquot of ACK lysed blood was labeled with 1 μM of Fluo-4-AM. Next, 300 μL of Fluo-4-AM labeled cell suspension were measured using a CytoFlex cytofluorimeter, 30 µL of 1 μM Ca-ionophore or 30 µL of 1 μM fMLP were added 20 s after the the measurement was started, and data were collected for 5 min. (**A**) Intracellular Ca^2+^-mobilization was measured by flow cytometry (whole blood, granulocyte gate) and the integral of the area under the curve was calculated. (**B**) Maximal signal intensities were calculated from intracellular Ca^2+^-mobilization measurement with flow cytometry (whole blood, granulocyte gate). Fluo-4-AM-stained NGs in 2 × 10^5^ cells/well concentration were seeded in a 96-well black V-shaped bottom plate. We used 0.1 μM of fMLP, 1 μM of Ca-ionophore (Calcimycin), 20 μM and 2 μM of BK in HBSS as treatments in 3-min kinetic measurement in three parallels. (**C**) Intracellular Ca^2+^-mobilization was measured with a microplate reader (isolated neutrophil granulocytes) and the integral of the area under the curve was calculated (3 parallels/subject). (**D**) Maximal signal intensities were calculated from intracellular Ca^2+^-mobilization measurement with a microplate reader (isolated neutrophil granulocytes, 3 parallels/subject). NGs of patients are indicated by red dots; NGs of healthy controls are indicated by blue dots. BK= bradykinin; fMLP= *N*-formylmethionyl-leucyl-phenylalanine. We used Mann–Whitney test (**A**, **B**) and Two-way ANOVA (**C**, **D**) for statistical analysis.

NGs are a very sensitive cell type, and NG preparation can easily modify neutrophil functions. Rapid RBC depletion of EDTA-anticoagulated blood lacking multiple centrifugation steps may preserve neutrophil functions better than other methods involving multiple centrifugation steps. Therefore, to verify that NGs preserved their function after preparation, we performed Ca^2+^-mobilization measurements on both RBC-depleted blood and purified NGs using flow cytometry and fluorescence plate reader, respectively. As the intracellular Ca^2+^-mobilization effect of fMLP measured by flow cytometry was very similar to that observed in purified NGs measured by fluorescence plate reader (Fig. 2A,B,C,D), the purified NG preparations seemed to be functional.

After validating the function of purified NGs, we used a fluorescence plate reader to detect Ca^2+^-mobilization in isolated NGs. fMLP induced similarly high Ca^2+^-mobilization (normalized to the response of Ca-ionophore (Calcimycin), 1 μM) in NGs from patients and controls. BK in 20 μM concentration induced a significant (treatments *p* < 0.0001) but more moderate Ca^2+^-mobilization in both study groups than in fMLP (Fig. 2C,D), but there was no difference in Ca^2+^ -mobilization between NGs of patients and controls.

### NG activation markers and activatablity

**Figure 3:**
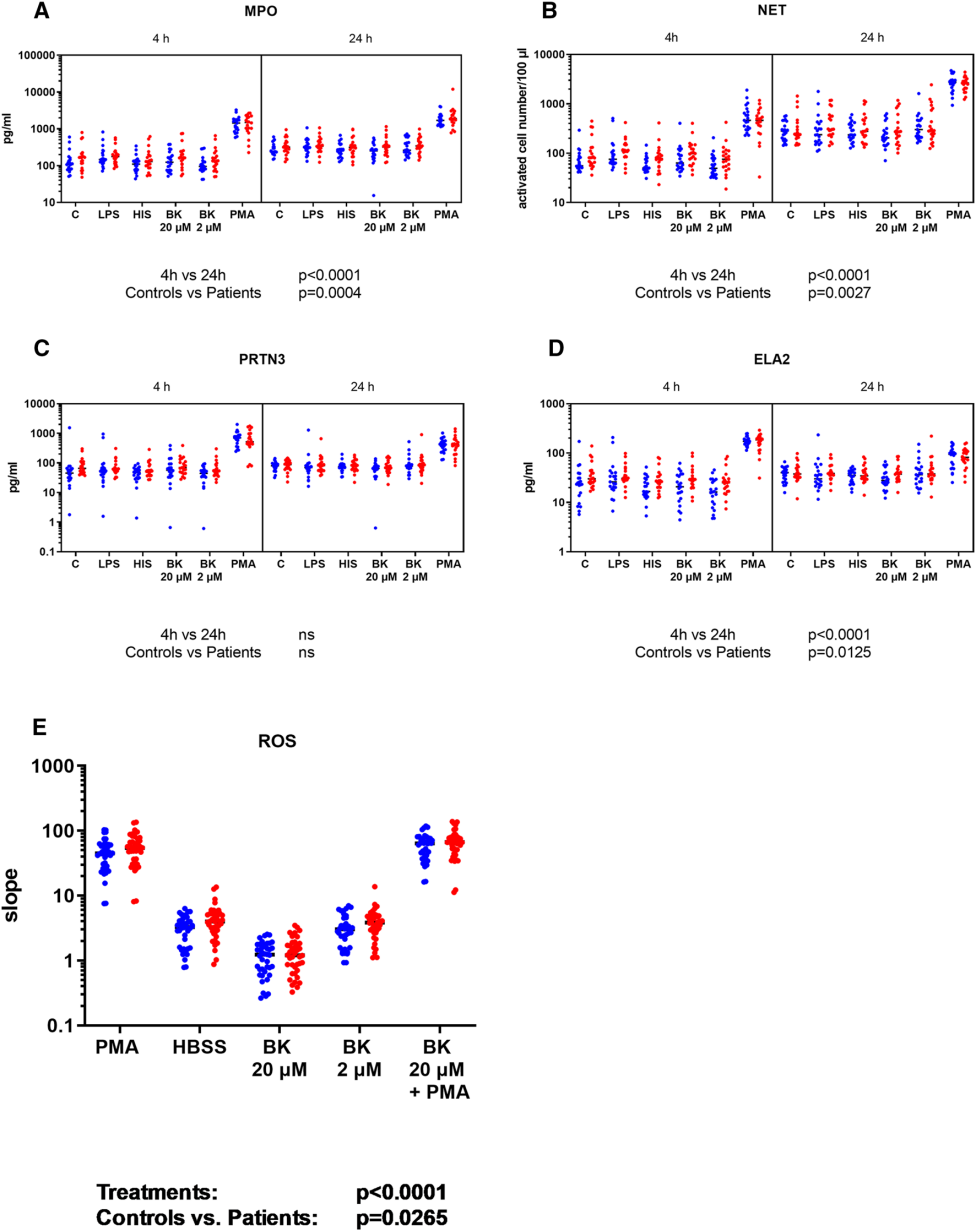
Activation state and activatability of neutrophil granulocytes isolated from patients and from healthy controls. Levels of (**A**) myeloperoxidase (MPO), (**B**) neutrophil extracellular trap (NET), (**C**) proteinase 3 (PRTN3), and (**D**) neutrophil elastase (ELA2) produced by isolated neutrophils, (**E**) ROS production by neutrophil granulocytes were measured. For panels **A**, **B**, **C** and **D**, 5 × 10^5^ NGs/well were seeded into 96-well U-shaped bottom plates. Cells were treated with 100 ng/ml of LPS, 50 μM of HIS, 2 μM and 20 μM of BK or 100 nM of PMA in 200 μl of RPMI medium. Cells were incubated at 37 °C in a CO_2_ incubator for 4 or 24 h. After incubation, the supernatant was collected and centrifuged at 10,000 g for 10 min to settle cell debris. The collected supernatant was aliquoted and stored at − 80 °C until use. For panel E, 0.5 × 10^5^ NGs/well were seeded in a 96-well black V-shaped bottom plate. We used 100 nM of PMA, 20 μM and 2 μM of BK, and 100 nM of PMA + 20 μM of BK together dissolved in HBSS as treatment. As substrate, we used 50 μM of Amplex Ultra Red (AUR) + 0.2 U/ml of horseradish peroxidase/well in a 25-min kinetic measurement in two parallels. The slope of the kinetic curve (calculated with GraphPad Prism v9.1.2 by linear regression) was proportional to the H_2_O_2_ produced by NGs. NGs of patients are indicated by red dots; NGs of healthy controls are indicated by blue dots. C = control, LPS = lipopolysaccharide, HIS = histamine, BK = bradykinin, PMA = phorbol 12-myristate 13-acetate. We used Three-way ANOVA (**A**, **B**, **C**, **D**, **E**) for statistical analysis.

We examined the basic activation state and activatablity of NGs from patients and controls. We measured activation markers of NGs in cell supernatants and found that NGs from patients produced significantly higher MPO, ELA2 and NET compared to NGs from controls (Fig. 3A,B,C,D, controls vs. patients *p* = 0.0004, *p* = 0.0125, *p* = 0.0027, respectively). Regardless of their origin, cells produced significantly higher levels of MPO, ELA2 and NET during 24 h of treatment than 4 h of treatment (4h vs. 24h *p* < 0.0001, *p* < 0.0001, *p* < 0.0001, respectively). We also measured ROS production of NGs. Although the distinct stimuli resulted in quite different ROS production (i.e. increased by phorbol 12-myristate 13-acetate (PMA) whereas decreased by BK), we found that NGs from patients produced more ROS than NGs from controls, either in the presence or absence of any treatment (Fig. 3E, controls vs. patients *p* = 0.0265).

### Adhesion of NGs to endothelium

**Figure 4:**
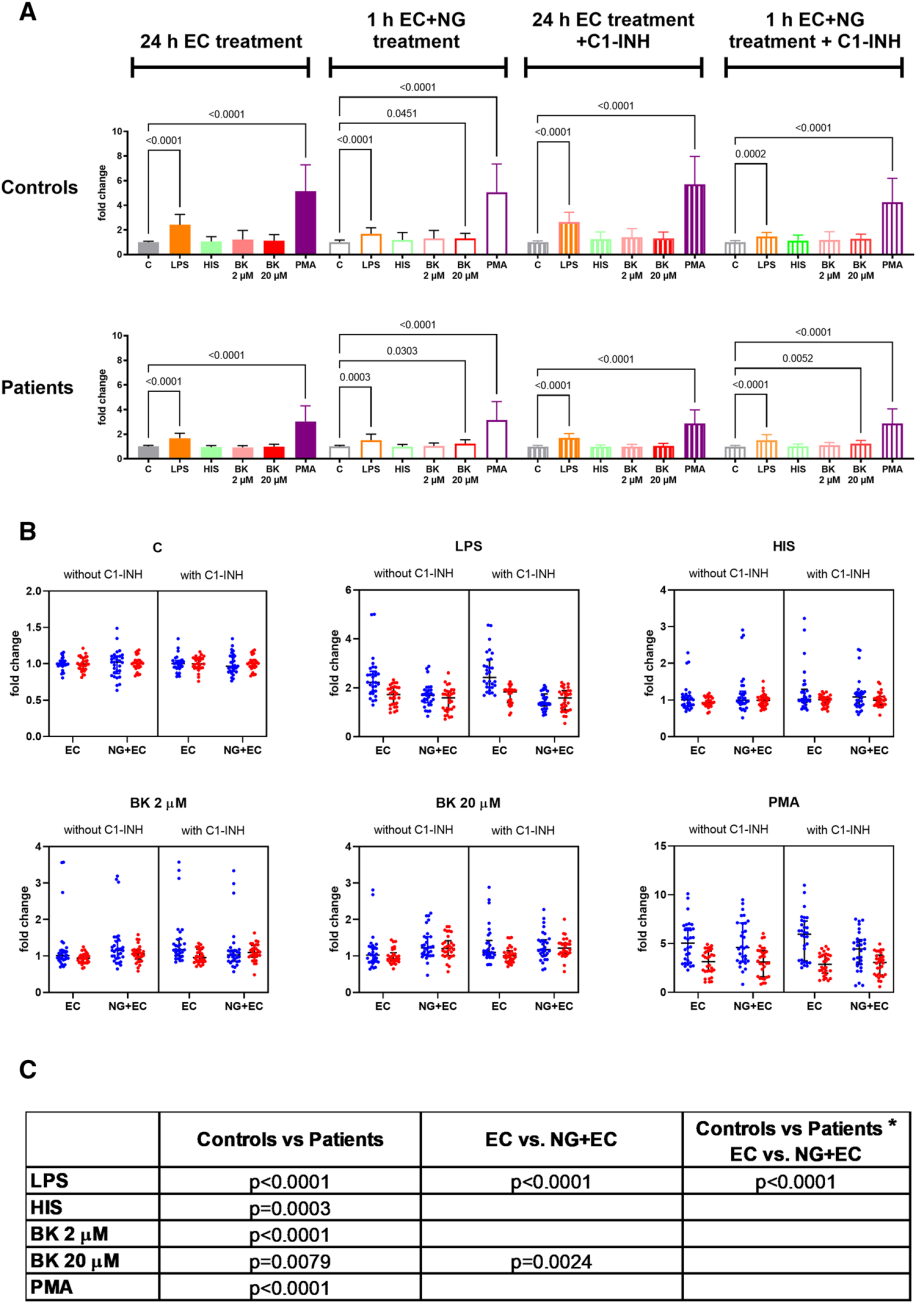
Adhesion of neutrophil granulocytes to endothelial cells. We grew human umbilical vein endothelial cells (HUVECs) in 96-well plates at 100% confluence. We used 100 ng/ml of LPS, 50 μM of histamine, 20 μM or 2 μM of BK or 100 nM of PMA as treatment in the presence or absence of C1-INH (200 μg/ml). We treated HUVECs for 24 h or co-treated HUVECs and NGs for 1 h. We labeled 17 × 10^6^ NGs with Oregon green dye (2 μg/ml). We added 0.5 × 10^6^ Oregon Green stained NGs/well to the HUVEC covered plate. After co-incubation of the two cell types for 1 h, non-adherent NGs were removed using an automated pipetting system (EpMotion 5070, Eppendorf SE automated pipetting device), and the residual fluorescence signal of the Oregon Green labeled NGs was measured. (**A**) Four adhesion assessing conditions were established according to the pre-stimulation and the presence of C1-INH. In each group, adhesion in response to different stimuli was normalized to its own unstimulated control. Relative mean (+/− SD) adhesion values from patients and controls are presented, and one-way ANOVA statistical tests were performed. (**B**) To show the effects of each stimulus according to the source of NGs (patients or controls), the presence or absence of C1-INH and the cells treated (i.e. ECs for 24 h or NGs and ECs co-treated for 1 h), a scatter plot format was used, and a three-way-ANOVA analysis was performed. Patients are indicated by red dots; healthy controls are indicated by blue dots. (**C**) The table summarizes the significant p values of the three-way ANOVA. C = control, LPS = lipopolysaccharide, HIS = histamine, BK = bradykinin, PMA = phorbol 12-myristate 13-acetate.

We also examined whether we could find any difference between the adhesion property of NGs from patients or controls. As adhesion was assessed according to four variables: (a) origin of NGs: controls vs patients, (b) presence vs. absence of C1-INH, (c) treatment of ECs vs co-treatment of ECs and NGs, and (d) different activation stimuli, we consider these results as mean +/− SD values in Fig. 4A. For simplicity, all p values are shown in Fig. 4A,C. We found that both lipopolysaccharide (LPS) and PMA significantly increased NG adhesion to ECs, irrespectively of the origin of neutrophils, presence of C1-INH and the cell type treated. Interestingly, 20 μM of BK only moderately increased adhesion when ECs and NGs were co-treated for 1 h (Fig. 4A). To further analyze adhesion properties, we grouped adhesion data by stimulus (variable d), performing a three-way ANOVA using the other three variables (variables a, b and c) (Fig. 4B). We found that NGs from patients attached less to ECs than NGs from controls for all stimuli (variable a). More adherent NGs were found when only ECs were treated with LPS for 24 h than when ECs and NGs were co-treated for 1 h (variable c). Interestingly, treatment with 20 μM of BK resulted in more adherent NGs when ECs and NGs were co-treated for 1 h than when only ECs were treated for 24 h (variable c). We found a significant interaction between the origin of NGs and the target cell type of treatment only for LPS treatment (variable a ↔ c)(Fig. 4C). Surprisingly, the presence or absence of C1-INH did not influence adhesion between ECs and NGs (variable b).

## Discussion

When the pathogenesis of angioedema (especially C1-INH-HAE) was started to be investigated a few decades ago, C1-INH and BK became the focus of research. Later, ECs turned out to have the utmost importance in the pathogenesis^7–9^. Although extensive investigations have revealed many details about this disease, several important questions have still remained unanswered, such as elevated NG count and activated NG status in C1-INH-HAE patients.

In the current study, we observed a trend towards elevated neutrophil counts in C1-INH-HAE patients, similar to our previous results^4–6^; however, the difference was not statistically significant. One possible reason for this discrepancy is that we performed NG counting after a multistep neutrophil isolation protocol. This process caused a significant variance in the measured NG count. The second reason is low sample size. In our current study, we performed extensive in vitro assays, which allowed us to include only a limited number of patients and controls, which reduces the power of the statistics.

Tak et al.^10^ and Summers et al.^11^ summarized several distinct pathophysiological processes that may underlie elevated NG counts. First, high NG counts could be the consequence of an elevated rate of neutrophil generation in the bone marrow and/or the elevated mobilization of neutrophils from the bone marrow. Our flow cytometry-based results showed that neutrophil maturation was normal in C1-INH-HAE patients.

Circulatory half-life and clearance of NGs from circulating and marginated pools may also affect NG count. Senescent NGs are readily taken up by macrophages in the spleen and the liver, in which CXCR4 and P-selectin regulated homing and phosphatidylserine induced phagocytosis via Annexin V are key components^11^. CXCR4 and P-selectin expression as well as Annexin V binding capacity were similar in NGs isolated from C1-INH-HAE patients and healthy controls; furthermore, there was no difference in spontaneous cell death as assessed by 7AAD incorporation, suggesting that clearance of NGs seems to be intact in C1-INH-HAE patients.

Although the marginated pool of NGs may comprise almost 50% of intravascular NGs^11^, it is not easy to study this fraction. We found no differences between the expression of adhesion molecules and chemokine receptors on the NGs of C1-INH-HAE patients compared to the expression of them on the NGs of healthy controls; however, the expression of partner receptors/ligands by ECs was certainly not assessed.

When we examined the activation status of NGs in the absence of any stimuli, NGs from patients were found to be more activated than those from controls, which was in complete accordance with our previous studies^5,6^. Ehrenfeld et al.^12^ reviewed the effect of kinins on NGs during inflammation, and found that the stimulation of BK receptor B1 causes a rapid activation of the ERK1/2 and p38 mitogen-activated protein kinases (MAPKs), leading to exocytosis of NG activation markers (e.g. MPO). Interestingly, in contrast to the above citations, the stimulation with BK had no effect or even inhibited NG activation in our study, although 20 µM of BK induced an expected intracellular Ca^2+^-mobilization. In a recent study, we described elevated kallikrein/C1-INH complexes in C1-INH-HAE patients (during symptom-free periods) compared with healthy controls and also at the onset of HAE attacks (kinetic follow-up period)^13^. It is also well established that the activation of KKS can occur on the surface of NGs and NET^5,14,15^. Considering the effect of BK and KKS enzymes on NGs and the effect of NG activity on KKS, we cannot decide whether NGs or their NET products induce KKS activation during HAE attacks, or, conversely, the activation of KKS attracts and activates NGs, but these results suggest an amplification loop between the activation of NGs and KKS. The background of this challenging interaction requires extensive further investigations.

We found very similar results when we tested the adhesion properties of NGs to ECs with our newly developed adhesion assay: interestingly, BK had no or just a negligible effect on NG adhesion to ECs.

In contrast to this minor effect of BK on cell adhesion, unstimulated NGs from patients attached less to the surface of ECs than those from controls. When we used LPS or PMA stimuli on ECs, the adhesion difference between the surface-attached NGs from C1-INH-HAE patients and from controls was much more striking. Moreover, the difference in adhesion properties was not caused by any C1-INH inhibitable serine protease released by NGs or the C1-INH itself, as C1-INH had no effect on adhesion. Our findings are in contrast to the results of Cai et al.^16^ who found in an in vitro system that C1-INH can bind E- and P-selectins on HUVECs surface and, through this interaction, it can prevent leucocyte adhesion. The different study setup may explain this contradiction, e.g. Cai et al. used HBSS puffer during incubation, whereas we used MCDB media with FBS content.

Given that in our functional adhesion experiments, we found no difference in adhesion strength when we treated ECs alone or when we co-treated ECs and NGs, and that the pattern of surface adhesion molecules of NGs was very similar in C1-INH-HAE patients and in controls, we can anticipate that treatments affected mostly the adhesion properties of ECs and not those of NGs. It would suggest that either ECs or the plasma of C1-INH-HAE patients carry a factor that changes EC/NG adhesion. One factor could be the soluble E-selectin (sE-selectin), a shed adhesion molecule of ECs. The potential role of sE-selectin may also be supported by our previous findings: we described elevated sE-selectin concentrations in the plasma of C1-INH-HAE patients^17^, which was further elevated during attacks^18^ and can act as a decoy molecule for E-selectin ligands. The different coverage of adhesion molecules involved in the attachment of NGs to the vessel wall may cause a biased NG ratio between marginated and freely circulating pools in C1-INH-HAE patients. This hypothesis of altered adhesion between ECs and neutrophils was further supported by our previous in vitro adhesion experiments, where the adhesion of dPLB-985 cells (which are differentiated neutrophil-like cells) to ECs were totally inhibited by sE-selectin^19^. Furthermore, Ruchaud-Sparagano et al.^20^ found that sE-selectin could increase ROS production of NGs, and we also found that ROS production was increased in NGs from patients compared to those from healthy controls.

Our study has several limitations. First of all, the sample size is very small (C1-INH-HAE is a rare disease and most of the patients use several prophylactic therapies), although our study population is quite large and is more closely matched for age and sex than other studies in the field C1-INH-HAE. Another limitation is that our study only investigated NGs but not ECs in the cell adhesion part of the study. This is because there is no method to obtain endothelial cells from C1-INH-HAE patients. Furthermore, due to the retrospective nature of our study, we cannot include some additional controls and more in-depth analyses (such as neutrophil ACE expression or use of icatibant, a potent BK receptor 2 antagonist).

## Conclusion

Our results, together with the lack of evidence of chronic inflammation in the C1-INH-HAE patients^21^, suggest that the high NG counts in these patients may be caused by disturbed adhesion to ECs biasing the ratio between marginated and circulating pools, whereas only a mild activation of circulating NGs occurs as a consequence of more active plasma serine proteases and/or from the elevated soluble E-selectin levels. It is largely unknown if this mild activation of NGs has positive or negative consequences for the C1-INH–HAE patients, but it is worth further investigation.

## Methods and patients

### Patients

Twenty C1-INH-HAE patients treated and followed up at the Hungarian Angioedema Center Reference and Excellence (Budapest, Hungary) and 21 age- and gender-matched healthy controls were enrolled in the study. Diagnosis of C1-INH-HAE patients was based on family history, medical history of the patient, physical examination and two complete complement laboratory tests [total classic complement cascade, C3, C4, C1-INH concentration level and C1-INH functional activity, anti-C1-INH antibodies (IgA, M, G)] performed 1–2 months apart. Genetic analysis of the *SERPING1* gene was performed in all cases. Whole blood samples from controls and attack-free C1-INH-HAE patients were collected in EDTA-containing tubes (3 mL) and 5 ml PBS + 100 μl heparin-sodium (25,000 IU) containing centrifuge tube (35–45 ml) between 01/09/2020 and 30/06/2021. C1-INH-HAE patients had not taken C1-INH concentrate or icatibant within 5 days before sample collection. Healthy controls were not taking any drugs and were not under any medical treatment. All subjects gave informed consent in accordance with the Declaration of Helsinki. The study protocol was approved by the Hungarian Regulatory Authority (BPR/021/03928–8/2014). Detailed demographic data of patients and controls are summarized in Table 1.

**Table 1:** Basic characteristics of the study population.

ROS, Ca^2+^, FACS, ELISA measurements	Patients (n = 20)	Controls (n = 21)
Age in years (mean; min–max)	43.6 (23–69)	42.9 (21–68)
Sex (women/men)	11/9	12/9
C1-INH-HAE type I/II	19/1	-

NG adhesion measurements	Patients (n = 10)	Controls (n = 10)
Age in years (mean; min–max)	41.5 (23–64)	41.1 (20–68)
Sex (women/men)	6/4	6/4
C1-INH-HAE type I/II	9/1	–

### Neutrophil granulocyte isolation

We collected 35–45 ml of blood in a 50-ml centrifuge tube containing 5 ml of PBS + 100 μl of heparin-sodium (25,000 IU) with an 18G needle without the use of vacuum. We added 5 ml of 6% dextran (Dextran from *Leuconostoc spp*. Mr 450,000–650,000, Sigma-Merck) and mixed it with blood. After sedimentation for 15–30 min, the plasma fraction was pipetted into a new centrifuge tube and centrifuged at 1500 rpm, at RT for 5 min. The supernatant was aspirated, and 5 ml of HBSS-Prep (HBSS without Ca^2+^ and Mg^2+^ supplemented with 20 mM of HEPES) was added, and the cells were gently mixed. The cells were layered on top of 5 ml of Ficoll (GE 17-1440-02, Merck KGaA, Germany), then centrifuged at 2500 rpm, at RT for 30 min. The supernatant was aspirated, 10 ml of HBSS-Prep was added, then the cells were centrifuged (5 min, RT, 1500 rpm). After aspiration of the supernatant, 5 ml of 0.2% NaCl was added for red blood cell (RBC) lysis for 60 s, then 5 ml of 1.6% NaCl was added to stop the lysis. The cells were centrifuged (5 min, RT, 1500 rpm). The supernatant was aspirated, and the cells were washed twice with 10 ml of HBSS-Prep. After the second centrifugation, 1 ml of HBSS-Prep was added, and the cells were counted. We changed the HBSS-Prep buffer immediately before the cells were used in different experiments.

### Reactive oxygen species (ROS) production measurement

We used 0.5 × 10^5^ NGs/well in a 96-well black V-shaped bottom plate. We used 100 nM of PMA, 20 μM and 2 μM of BK, and 100 nM of PMA + 20 μM of BK together dissolved in HBSS as treatments. As substrate, we used 50 μM of Amplex Ultra Red (AUR) + 0.2 U/ml of horseradish peroxidase /well in a 25-min kinetic measurement in two parallels. We calculated the slope for each well in GraphPad Prism v9.1.2 (GraphPad Software Inc.) by linear regression fitted to the kinetic measurement data and used the calculated slope as the value of ROS production.

### Intracellular Ca^2+^-mobilization measurement by microplate reader

We used 2 × 10^5^ NGs/well stained with Fluo-4-AM (F14217 Invitrogen, Thermo Fisher Scientific, 1:500) in a 96-well black V-shaped bottom plate. We used 0.1 μM of fMLP, 1 μM of Ca-ionophore (Calcimycin, Calcium Ionophore A23187 hemimagnesium salt, C9400, Sigma-Aldrich), 20 μM and 2 μM of BK in HBSS as treatments in 3-min kinetic measurement in three parallels. Ca-ionophore was used as a positive control, and fluorescence signals caused by other agents were presented as the percentage of the fluorescence signal caused by Ca-ionophore.

### Neutrophil granulocyte adhesion to endothelial cells

We grew Human Umbilical Vein Endothelial Cells (HUVECs) in 96-well plates at 100% confluence (as a model of the vessel wall). We used 100 ng/ml of LPS, 50 μM of histamine (HIS), 20 μM or 2 μM of BK or 100 nM of PMA as treatment in the presence or absence of C1-INH (200 ug/ml). We treated HUVECs for 24 h or co-treated HUVECs and NGs for 1 h. We labeled 17 × 10^6^ NGs cells with Oregon green dye (CellTrace™ Oregon Green™ 488 carboxy-DFFDA, SE, Invitrogen, Thermo Fisher Scientific, 1 mg/ml) in 1:500 dilution (HBSS-Prep) and incubated them at 4 °C for 20 min. Then, we centrifuged the cells at 1500 rpm for 5 min. We incubated the cells in 5 ml of HBSS-Prep at 4 °C for 20 min, resuspended them in 1 ml of MCDB cell culture medium after centrifugation, and counted them. We added 0.5 × 10^6^ Oregon Green stained NGs/well to the HUVEC covered plate. After co-incubation of the two cell types for 1 h, non-adherent NGs were removed using an automated pipetting system (EpMotion® 5070, Eppendorf SE automated pipetting device providing uniform vacuum forces to each well), and the residual fluorescence signal was measured for Oregon Green labeled NGs.

### Supernatant collection

We seeded 5 × 10^5^ NGs/well into 96-well U-shaped bottom plates. Cells were treated with 100 ng/ml of LPS, 50 μM of HIS, 2 or 20 μM of BK, or 100 nM of PMA in 200 μl of RPMI media. Cells were incubated at 37 °C in a CO_2_ thermostat for 4 or 24 h. After incubation, the supernatant was collected and centrifuged at 10,000 g for 10 min to settle cell debris. The collected supernatant was aliquoted and stored at − 80 °C until use.

### Activation marker measurements from cell supernatant

To determine the levels of enzymes myeloperoxidase (MPO), neutrophil elastase (ELA2) and proteinase 3 (PRTN3), we used commercially available ELISA KITs from R&D Systems (DY3174, DY9167-05 and DY6134-05) according to the instructions of the manufacturer. To measure neutrophil extracellular traps (NETs), we used an in-house ELISA method published previously^5^.

Fluorescence (ROS-, intracellular Ca^2+^-mobilization-, and adhesion measurement) and OD measurements (ELISA) were performed using an Infinite® M1000 PRO microplate reader.

### Flow-cytometry measurements

EDTA-anticoagulated blood was lysed with Ammonium-Chloride-Potassium (ACK) red blood cell lysis buffer in the dark at room temperature for 10 min. After centrifugation, pellets were brought to the original blood volume with PBS, and 50 μL of aliquots were labeled with antibodies (for details see Supplementary Table 2) for 20 min, then washed, and 100,000 cells were measured using a Beckman Coulter CytoFlex® cytofluorimeter. A second aliquot of ACK lysed blood was labeled with 1 μM of Fluo-4-AM (ThermoFisher) for 30 min and equilibrated in HBSS for 30 min. Next, 300 μL of Fluo-4-AM labeled cell suspension were measured in a CytoFlex® cytofluorimeter, 30 µL of 1 μM Ca-ionophore or 30 µL of 1 μM fMLP were added 20 s after the measurement was started, and data were collected for 5 min. Ca-ionophore was used as a positive control, and the fluorescence signal induced by other agents was expressed as the percentage of the fluorescence signal induced by Ca-ionophore.

Data were evaluated using CytExpert 2.4 and Kaluza® C software. The percentage of positive and negative populations and/or mean fluorescence intensity values were compared between healthy controls and C1-INH-HAE population.

### Statistical analysis

Results of ELISA measurements were analyzed and interpreted using GraphPad Prism v9.1.2 (GraphPad Software Inc.). Statistical analysis was performed using D'Agostino & Pearson normality test, One-way ANOVA, Two-way ANOVA, Three-way ANOVA, and Mann–Whitney test for ROS measurement, intracellular Ca^2+^-mobilization measurement with a microplate reader, activation marker measurements from cell supernatant and NG adhesion for EC measurement.

As most distributions were non-normal, we used median values of fluorescence intensity. We calculated the ratio of median fluorescence intensity values for the given marker and its isotype control. We used these ratios and compared them (between controls and HAE patients) with Student’s t test in flow-cytometry measurements.

*P* < 0.05 was considered as statistically significant in each case.

### Supplementary Information

Supplementary Information.

## Data Availability

The datasets generated during and/or analyzed during the current study are available from the corresponding author on reasonable request.
