# Supplementary material for: Decreased adhesion to endothelium leads to elevated neutrophil granulocyte count in hereditary angioedema patients
Source: Sci Rep. 2023 Aug 17;13:13366. doi: 10.1038/s41598-023-40442-9 (PMC10435475; doi:10.1038/s41598-023-40442-9)
Supplement: Supplementary file 1 — Supplementary Information. [file 41598_2023_40442_MOESM1_ESM.pdf]

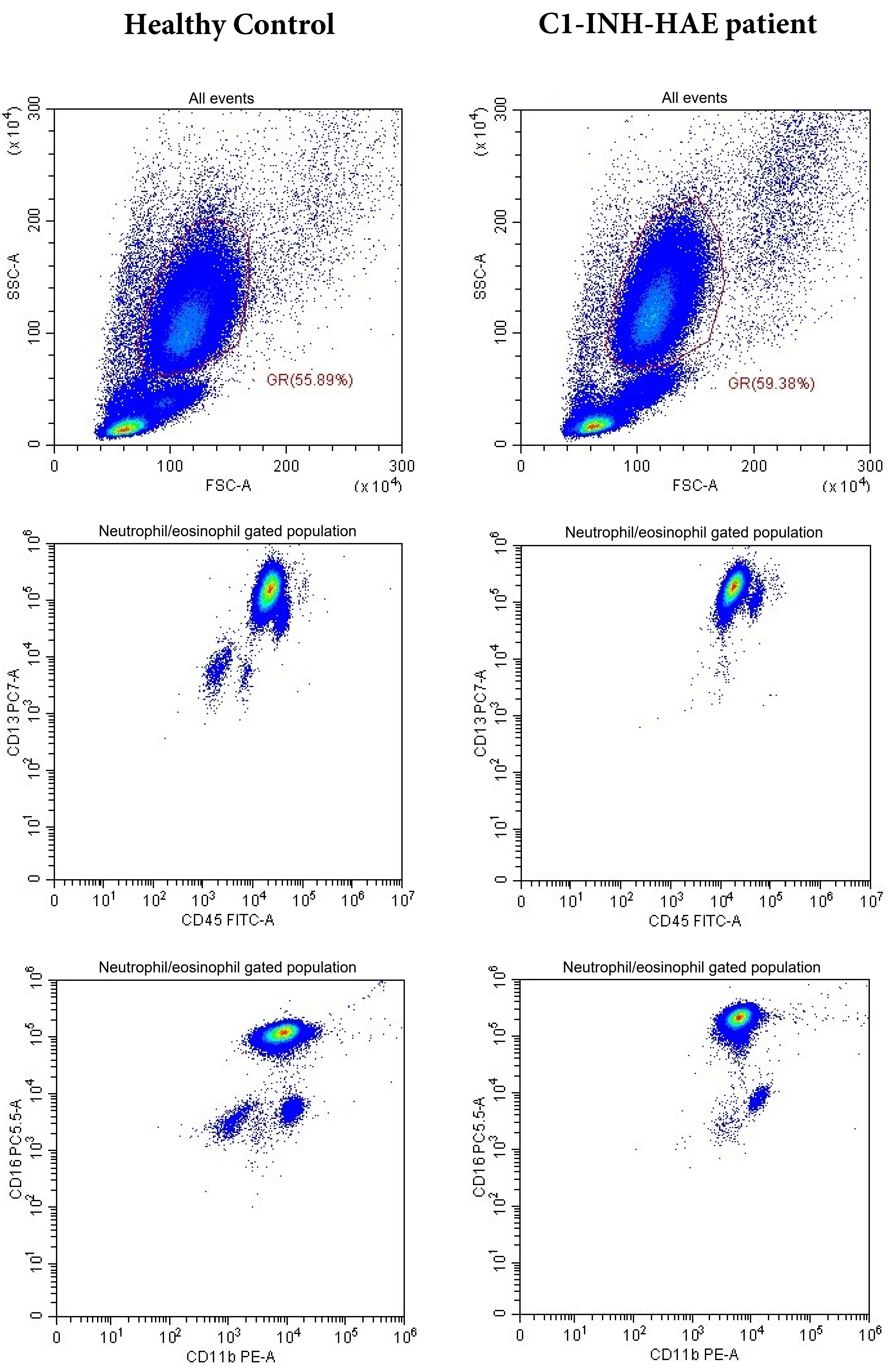
Healthy Control
C1-INH-HAE patient
All events
SSC-A
FSC-A
(x 10^4)
GR(55.89%)
GR(59.38%)
Neutrophil/eosinophil gated population
CD13 PC7-A
CD45 FITC-A
CD16 PC5.5-A
CD11b PE-A

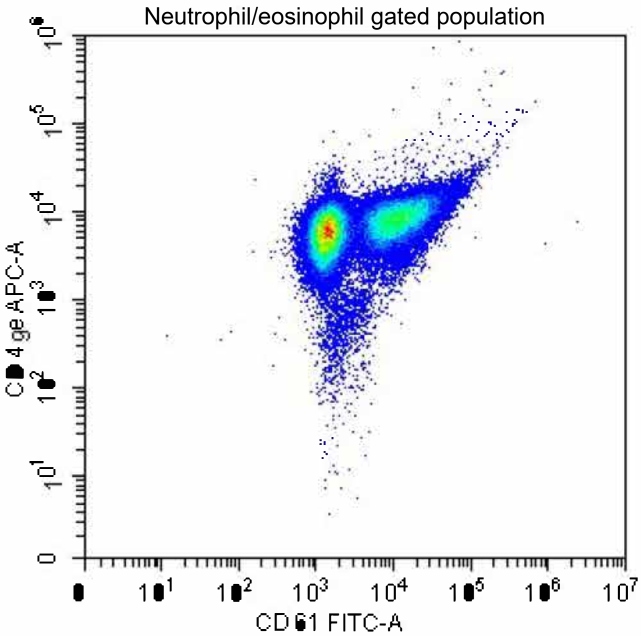
Neutrophil/eosinophil gated population
FITC-A

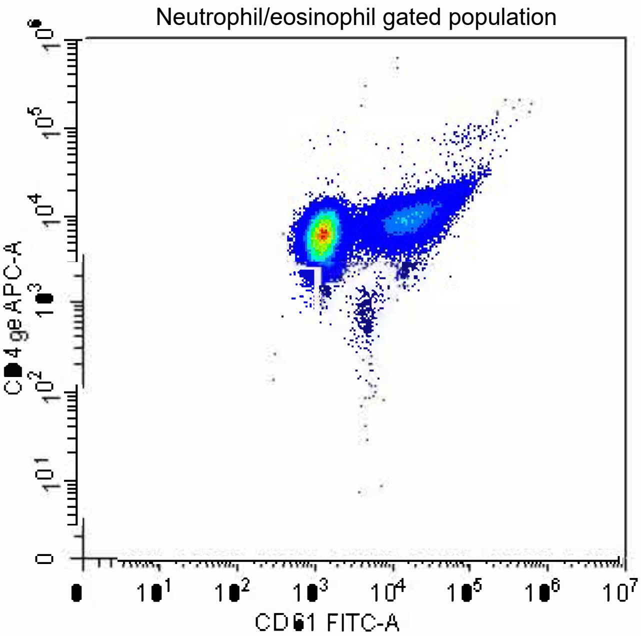
Neutrophil/eosinophil gated population
FITC-A

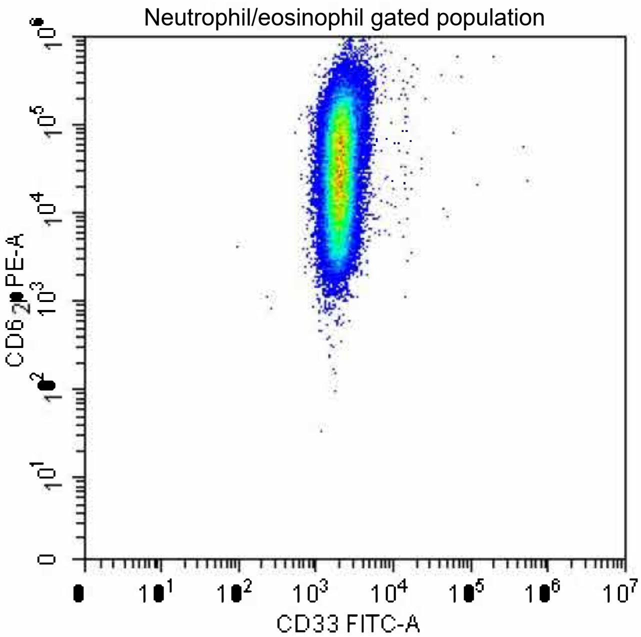
Neutrophil/eosinophil gated population
CD33 FITC-A

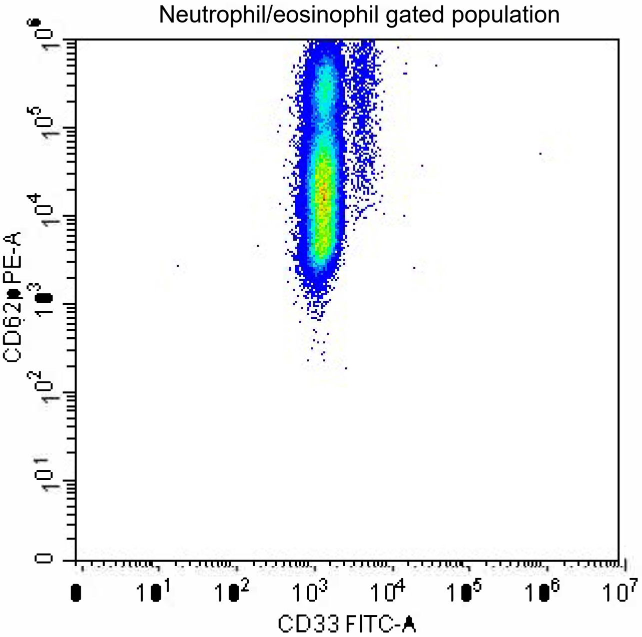
Neutrophil/eosinophil gated population
CD33 FITC-A

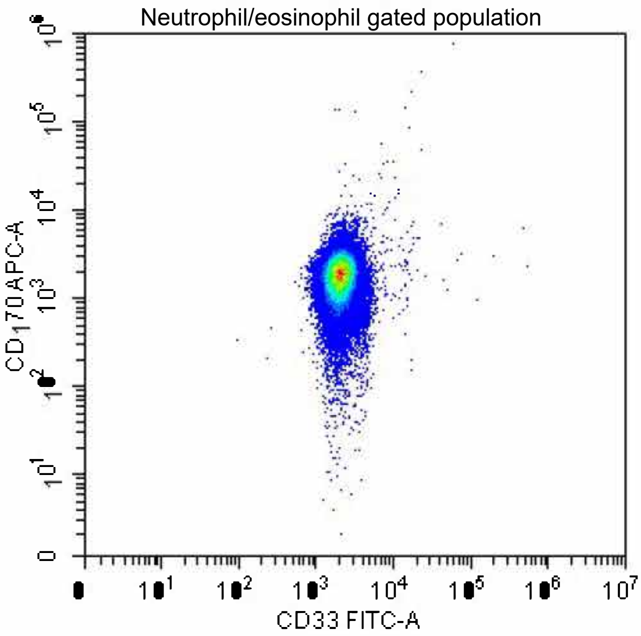
Neutrophil/eosinophil gated population
CD170 APC-A
CD33 FITC-A

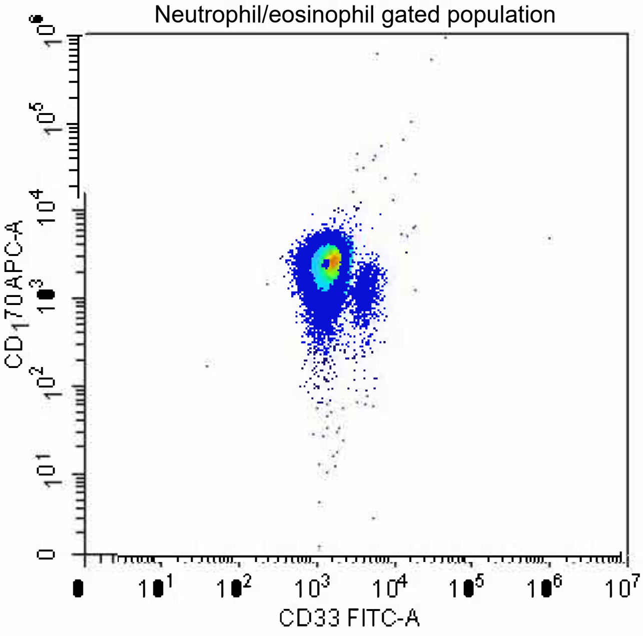
Neutrophil/eosinophil gated population
CD170 APC-A
CD33 FITC-A

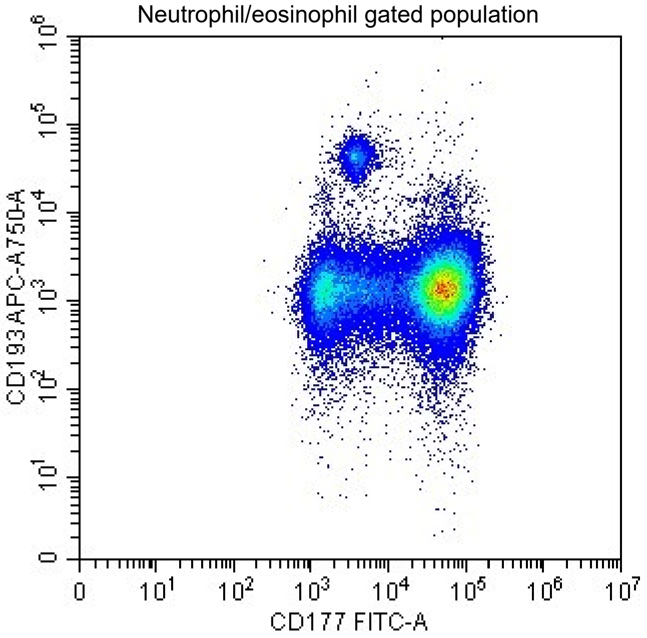
Neutrophil/eosinophil gated population
CD193 APC-A750-A
CD177 FITC-A

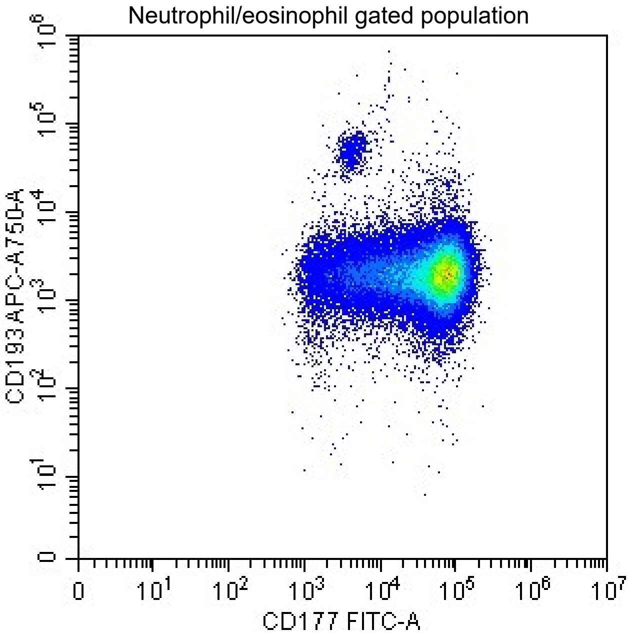
Neutrophil/eosinophil gated population
CD193 APC-A750-A
CD177 FITC-A

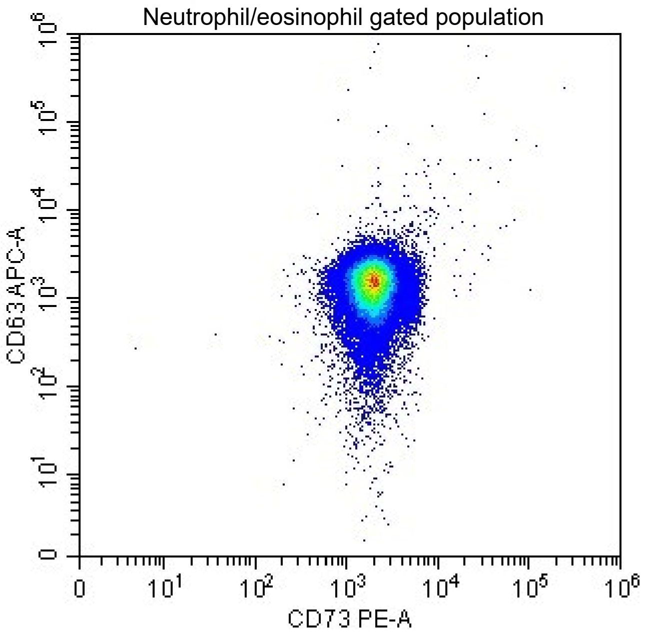
Neutrophil/eosinophil gated population
CD63 APC-A
CD73 PE-A

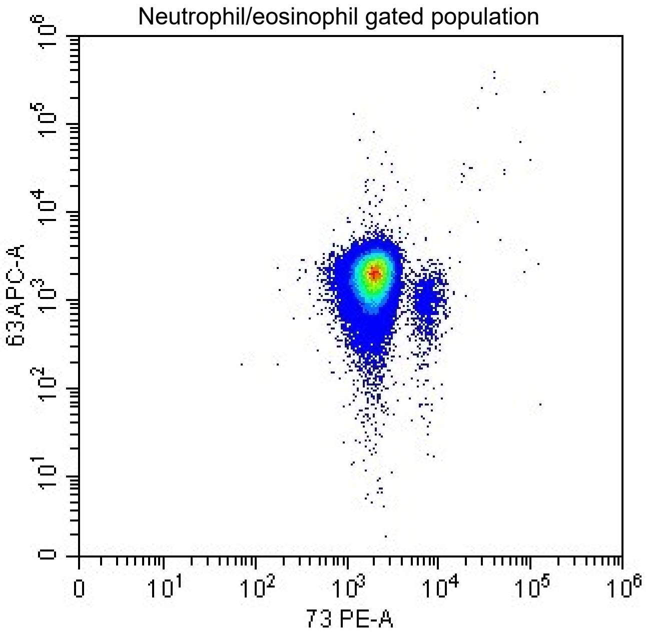
Neutrophil/eosinophil gated population
63 APC-A
73 PE-A

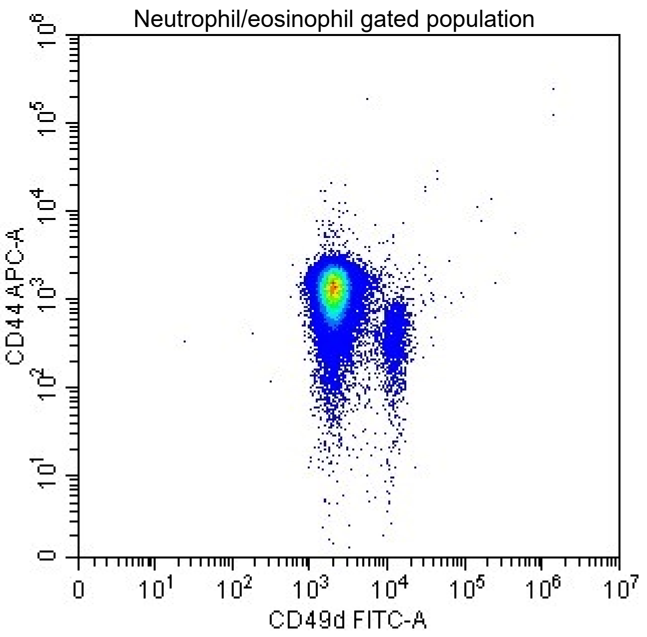
Neutrophil/eosinophil gated population
CD44 APC-A
CD49d FITC-A

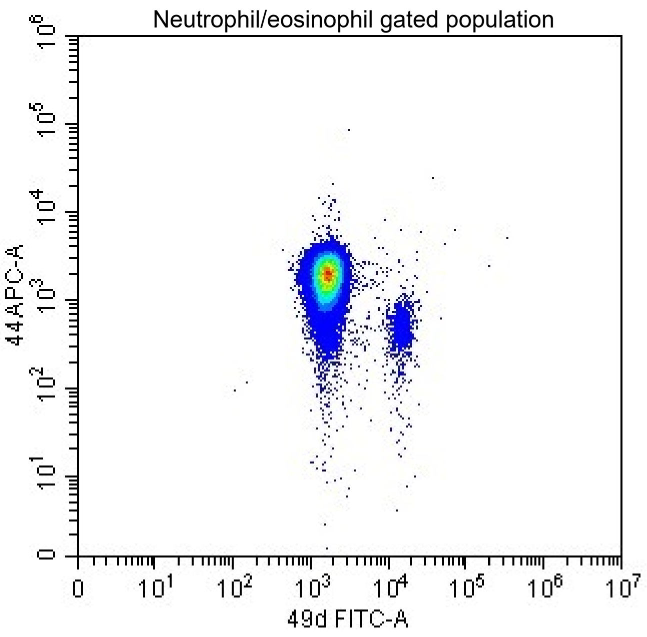
Neutrophil/eosinophil gated population
44 APC-A
49d FITC-A

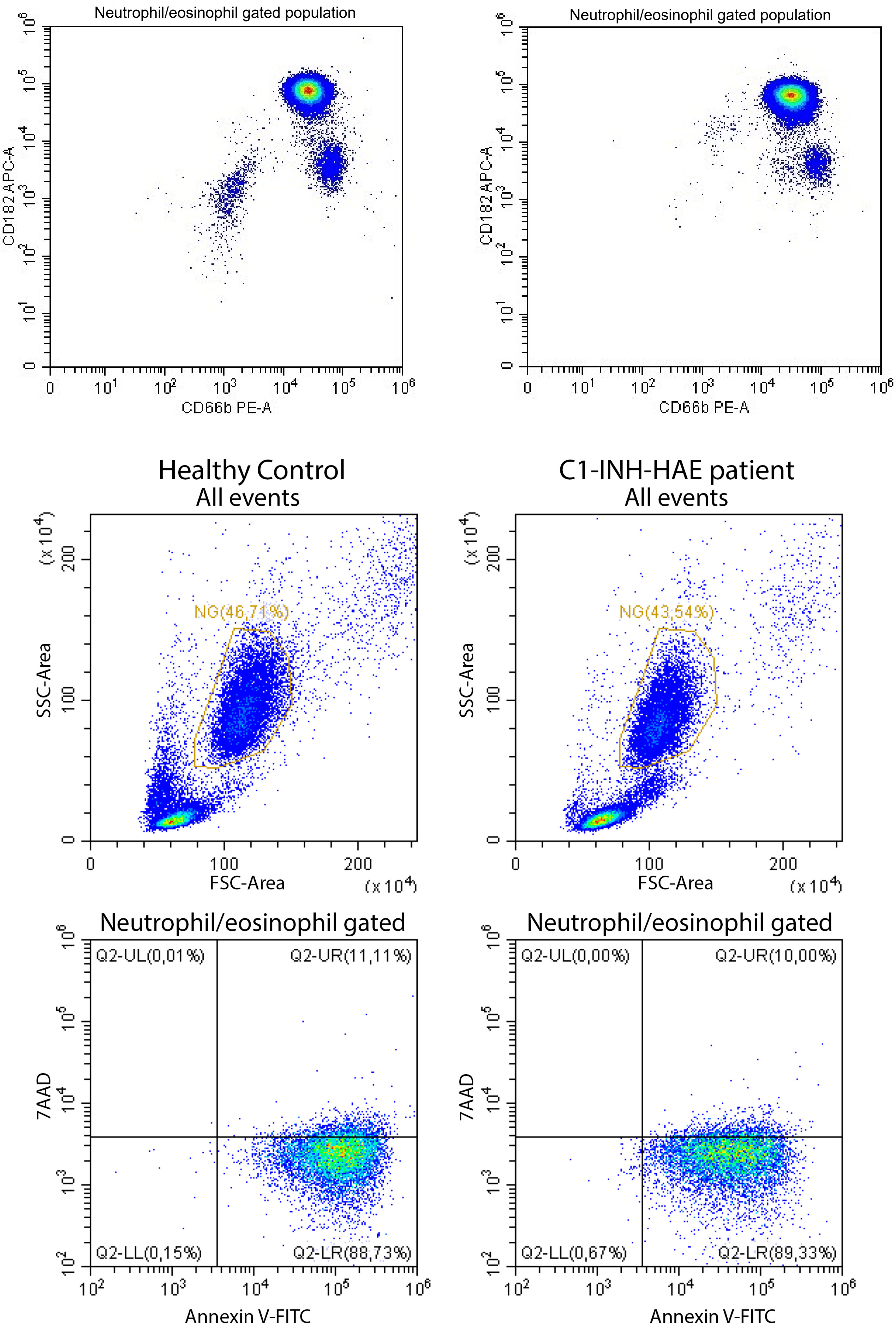
Neutrophil/eosinophil gated population
CD182 APC-A
CD66b PE-A
Neutrophil/eosinophil gated population
CD182 APC-A
CD66b PE-A
Healthy Control
All events
C1-INH-HAE patient
All events
NG(46,71%)
SSC-Area
FSC-Area
NG(43,54%)
SSC-Area
FSC-Area
Neutrophil/eosinophil gated
Neutrophil/eosinophil gated
Q2-UL(0,01%)
Q2-UR(11,11%)
Q2-LL(0,15%)
Q2-LR(88,73%)
7AAD
Annexin V-FITC
Q2-UL(0,00%)
Q2-UR(10,00%)
Q2-LL(0,67%)
Q2-LR(89,33%)
7AAD
Annexin V-FITC

Supplementary Figure 1. Distribution of NG cell surface markers and Annexin V staining. Pseudo color plots of representative pairs of C1-INH-HAE patient and matched healthy control are shown. Note that each panel (except FSC/SSC diagram) visualizes neutrophil/eosinophil gated population.

Supplementary Table 1. Median fluorescence intensity (MFI) of the CD markers measured on the surface of neutrophil/eosinophil granulocytes. We compared the isotype control normalized CD marker MFI values of the healthy controls and C1-INH-HAE patients with Student's t test.

	C1-INH-HAE MFI	Healthy control MFI	p value
CD11a	13324	14378	0.1899
CD11b	5691	6573	0.1665
CD11b	7403	8832	0.1153
CD13	144775	130711	0.5966
CD16	214133	195169	0.3064
CD16	205852	188282	0.2487
CD16	204888	188678	0.2382
CD16	205577	186306	0.3318
CD16	207375	184933	0.2279
CD33	1866	2119	0.2083
CD44	1739	1433	0.1567
CD45	25584	23476	0.8668
CD49b	2050	2221	0.5439
CD49d	2189	2409	0.0782
CD49e	7582	6333	0.2824
CD51	6317	5543	0.5439
**CD61**	**9259**	**6785**	**0.0071**
CD62p	77290	67539	0.5101
CD63	2231	1782	0.1491
CD66b	40569	29815	0.2083
CD66b	30323	21778	0.0738
CD73	2043	2107	0.4154
CD114	10457	11523	0.3721
CD116	5590	6226	0.2279
CD170	2654	2202	0.3189
CD177	24653	28698	0.2824
CD182	71785	63390	0.1990
CD184	8071	7644	0.2943
CD191	9683	9874	0.9897
CD192	862.9	717.8	0.1153
CD193	5928	6734	0.3584
CD193	3374	3811	0.4007
CD195	2321	2433	0.3721

Supplementary Table 2. Combinations of NG cell surface markers. To measure adhesion and maturation properties of NGs with flow cytometry. four-. five- or six-color labelling was used. Additional tubes with isotype controls were utilized to determine background fluorescence.

Tube number	FITC	PE	PerCP	PC7	APC	AC7
1	CD45	CD11b	CD16	CD13	CD66b	CD193
2	CD177	CD73	CD16	-	CD63	CD193
3	CD61	CD51	CD16	-	CD49e	-
4	CD49d	CD49b	CD16	-	CD44	-
5	CD11a	CD11b	CD195	CD191	CD192	-
6	CD116	CD66b	CD114	-	CD182	CD184
7	CD33	CD62p	CD16	-	CD170	-
